# How common is otogenic meningitis? A retrospective study in southern Sweden over 18 years

**DOI:** 10.1007/s15010-024-02195-z

**Published:** 2024-02-28

**Authors:** Nora Bjar, Ann Hermansson, Marie Gisselsson-Solen

**Affiliations:** 1 Vårdcentralen Lunden, Ystadgatan 53c, 214 44 Malmö, Sweden; 2 Department of Otorhinolaryngology, Head and Neck Surgery, Skåne University Hospital, 221 85 Lund, Sweden

**Keywords:** Bacterial meningitis, Acute otitis media, AOM, Complication, Pneumococcal conjugate vaccine, PCV

## Abstract

**Background:**

Bacterial meningitis is a rare, but life-threatening disease, which sometimes occurs as a complication to acute otitis media (AOM). The proportion of meningitis cases originating from AOM is not clear.

**Purpose:**

The aim of this study was to investigate the proportion of meningitis cases caused by AOM, to compare risk factors, bacteriology and outcome between otogenic and non-otogenic meningitis, and to analyse the incidence of bacterial meningitis after the introduction of conjugate pneumococcal vaccines (PCV).

**Methods:**

The medical charts of all patients admitted to hospitals in southern Sweden with bacterial meningitis between 2000 and 2017 were retrieved. Based on otoscopy and/or imaging, the proportion of otogenic meningitis cases was calculated, as were annual incidences.

**Results:**

A total of 216 patients were identified, 25 of whom died. The proportion of otogenic meningitis was 31% but varied from 6% among teenagers to 40% among adults. Before PCV, 23% of all meningitis cases were children < 2 years, compared to 1% post-PCV. The average incidence in the adult population, on the other hand, increased post-PCV, though there were large annual variations. *S. pneumoniae* was the most commonly identified pathogen in everyone but teenagers, in whom *N. meningitidis* was predominant.

**Conclusion:**

AOM is an important cause of meningitis in children and adults. Though bacterial meningitis almost disappeared in children < 2 years after the introduction of PCV, the incidence of pneumococcal meningitis in adults seems to have increased.

## Introduction

Despite advances in medical care, bacterial meningitis continues to be a challenging medical condition. Mortality rates vary greatly, depending on pathogen, and setting, however, is substantial also in countries with an advanced medical health care [1]. The risk of sequelae, such as cognitive impairments, hearing loss, or seizures, after recovery, is also substantial with approximately 20% of surviving patients developing at least one sequela [2].

Bacterial meningitis sometimes develops as a complication to acute otitis media (AOM); however, few studies have investigated the proportion of otogenic meningitis. AOM is one of the most common bacterial infections worldwide, and though most episodes are benign and resolve spontaneously, some lead to complications such as meningitis or mastoiditis [3, 4]. It has been shown that patients with bacterial meningitis and concurrent AOM have a more unfavourable outcome and a greater risk for post-meningitis hearing loss [5, 6].

The most common bacteria causing community-acquired meningitis are *Streptococcus pneumoniae* and *Neisseria meningitides* [1]. Previous studies have shown that pneumococcal meningitis is associated with a greater risk for severe outcomes [2, 5]. *S. pneumoniae* is also the most common pathogen in AOM and is particularly often associated with AOM with complications.

There are many knowledge gaps regarding bacterial meningitis. Studies aiming at finding out the proportion of cases occurring as a complication to AOM are very scarce, and though it seems clear that pneumococcal meningitis with vaccine serotypes have declined in young children after the introduction of pneumococcal conjugate vaccines (PCVs), there is conflicting evidence concerning pneumococcal meningitis incidence in adults, and there are also signs of an increased incidence of all-type pneumococcal meningitis due to replacement with non-vaccine serotypes.

The purpose of this study was to investigate what proportion of bacterial meningitis cases that were of otogenic origin in an 18-year retrospective regional cohort. Secondary outcomes were to investigate changes in incidence and bacteriology after the introduction of conjugate pneumococcal vaccination.

## Methods

In this retrospective, observational study, medical records from all patients admitted to hospitals in the county of Skåne, Sweden (population 1.3 million) with the ICD-code G00 (bacterial meningitis) between 2000 and 2017 were retrieved. Exclusion criteria were neonatal, viral, fungal or non-infectious meningitis, *Borrelia* or nosocomial, and postoperative or ventricular shunt-related infections. Information about gender, age, physical parameters on admission, otoscopy/otomicroscopy results, CT/MRI signs of middle ear infection, microbiological results, subjective hearing loss and hearing tests was extracted from medical charts. Population data were collected from Statistics Sweden. Pneumococcal conjugate vaccine was introduced in 2009 in the form of PCV7, which in 2010 was replaced by PCV10.

Hearing results were presented in a separate article [6].

Microbiological analyses were performed according to current clinical practice. Cultures were performed on cerebrospinal fluid, but in some cases also on blood, nasopharynx, and middle ear fluid. In addition, during the latter part of the study, a standard PCR for the most common meningitis pathogens was introduced.

Statistical analyses were performed using Excel and Stata 16.1 (Stata Corp, College Station, Texas, USA). The patients were categorised into five age groups: < 2 years, 2–11 years, 12–19 years, 20–64 years, and 65 years and above.

A high fever was defined as a temperature of 39 °C and above, sub-febrility as a temperature of 37.5°–38.9° and no fever as anything below 37.5 °C. Associations between categorical variables were analysed using uni- and multivariable logistic regression; retrieving odds ratios (OR), 95% confidence intervals (CI) and *p*-values. Variables that were associated with the outcome in the univariable analysis were then adjusted for in the multivariable analysis. Differences in proportions between two groups were compared using a Chi^2^-test.

The study was approved by the Ethics Review Authority. The STROBE guideline was used for the preparation of the manuscript.

The role of the funding source was limited to provide the researchers with research time. The funding sources played no part in planning or carrying out the study, nor in the analyses or preparation of the manuscript.

## Results

**Fig. 1:**
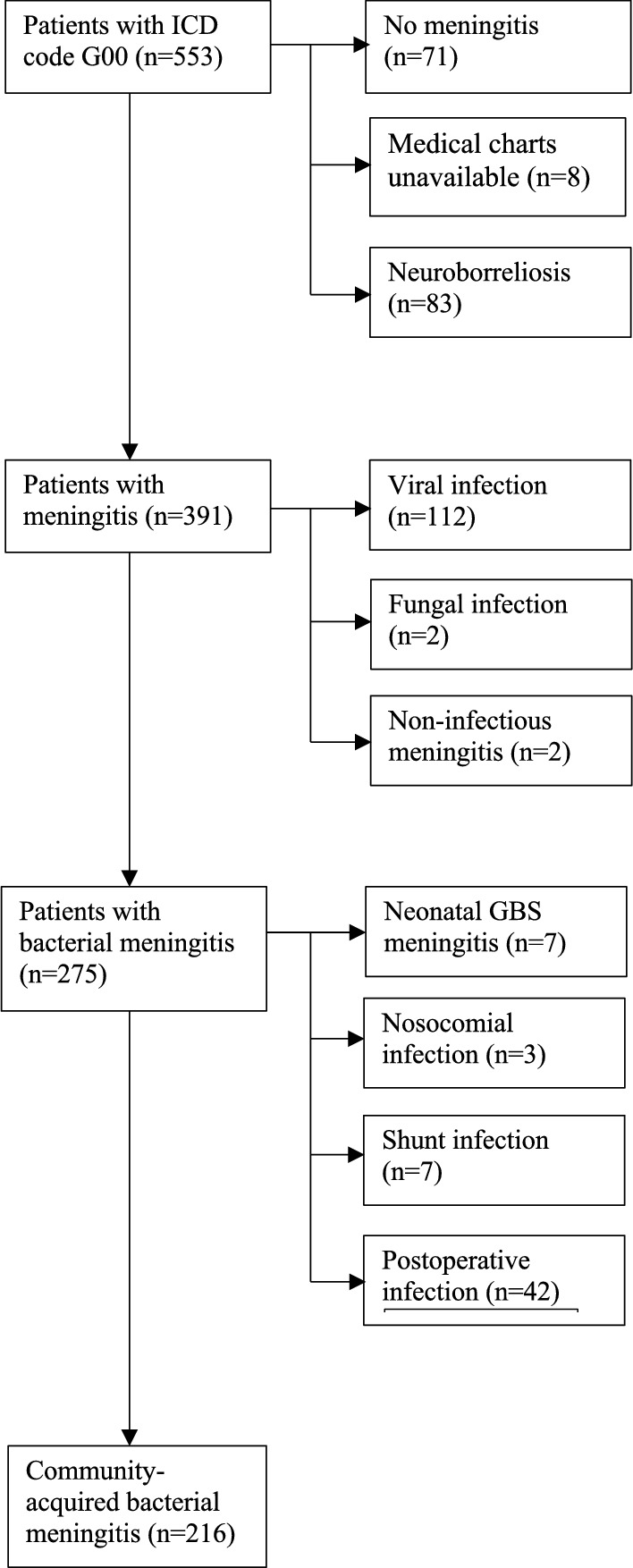
Study flow chart

Searches identified 553 patients with the diagnosis G00 during the 18-year study period. After applying exclusion criteria, 216 patients with community-acquired bacterial meningitis remained (Fig. 1). One hundred and nine patients (50%) were female. The mean age was 50 years; the youngest patient was three months and the oldest was 89 years. Forty-one (19%) of the patients were under the age of 18 years. For demographic and microbiological information, see Tables 1 and 2.

**Table 1:** Demographics of study patients

Demographic data		Number* (%)		
		Pre-PCV	Post-PCV	Total
Gender	Female	40 (55)	69 (48)	109 (50)
	Male	33 (45)	74 (52)	107 (50)
Fever	None	7 (11)	27 (21)	34 (17)
	Subfebrility	28 (42)	45 (35)	73 (37)
	High fever	31 (47)	57 (44)	88 (45%)
Headache	No	4 (12)	14 (18)	18 (16%)
	Yes	29 (88)	64 (82)	93 (84%)
Neck stiffness	No	20 (34)	45 (37)	65 (36)
	Yes	38 (66)	76 (63)	114 (53)
Affected consciousness	No	24 (34)	53 (38)	77 (36)
	Yes, but conscious	23 (33)	52 (37)	75 (36)
	Unconscious	23 (33)	36 (26)	59 (28)
Survived	Yes	66 (90)	125 (87)	191 (88)
	No	7 (10)	18 (13)	25 (12)
Pathogen	*S. pneumoniae*	39 (53)	79 (55)	118 (55)
	*N. meningitidis*	11 (15)	15 (10)	26 (12)
	*S. pyogenes*	3 (4)	2 (1)	5 (2)
	*S. aureus*	1 (1)	10 (7)	11 (5)
	*Listeria*	1 (1)	4 (2)	5 (2)
	*H. influenzae* type B	4 (5)	6 (4)	10 (5)
	Group B streptococci**	1 (1)	3 (2)	4 (2)

*Due to missing data in some cases, numbers do not always add up to 216

**Neonatal GBS infections were excluded

**Table 2:** Association between age and various outcomes, entire cohort; N (% of cases in the respective age group)

		Age group					
		< 2	2–11	12–19	20–64	≥ 65	Total
Number of patients		19	9	19	93	76	216
Otogenic meningitis		4 (21%)	3 (33%)	1 (6%)	37 (56%)	21 (32%)	66 (31%)
Pathogen	*S. pneumoniae*	16 (84%)	4 (44%)	2 (11%)	53 (57%)	43 (57%)	118 (55%)
	*N. meningitidis*	1 (11%)	3 (33%)	12 (63%)	8 (9%)	1 (1%)	25 (12%)
	*S. pyogenes*	0 (0%)	1 (11%)	0 (0%)	2 (2%)	2 (3%)	5 (2%)
	*S. aureus*	0 (0%)	0 (0%)	0 (0%)	6 (6%)	5 (7%)	11 (5%)
	*Listeria*	0 (0%)	0 (0%)	0 (0%)	1 (1%)	4 (5%)	5 (2%)
	*H. influenzae type B*	0 (0%)	0 (0%)	1 (5%)	3 (3%)	6 (8%)	10 (5%)
	GBS**	0 (0%)	1 (11%)	0 (0%)	1 (1%)	2 (3%)	4 (2%)
Died		2 (11%)	1 (11%)	1 (5%)	13 (14%)	8 (11%)	25 (12%)

Twenty-five of the patients died, yielding a mortality rate of 12%. Of the 25, eight had growth of *S. pneumoniae*, three of *S. aureus*, one of *Listeria monocytogenes*, and in 13 cases, the pathogen was unknown. At least 17 (68%) of the patients who died had an affected consciousness when they were admitted to hospital, and 12 (48%) were unconscious. This meant that the odds of dying were higher for unconscious patients (OR = 2.73; 95% CI = 1.17–6.40; *p* = 0.02).

In 79 cases (37%), an ENT surgeon had examined the patient, and an additional 37 patients had undergone otoscopy performed by another specialist. This meant that otoscopy/otomicroscopy was performed in 116 of the 216 cases (54%). Among the remaining 100 patients, 98 had undergone CT/MRI scans, meaning it was possible to determine whether the patient had a concurrent AOM in 214 of the 216 cases (99%). Fifty-five of the patients had a concurrent AOM according to otomicroscopy/otoscopy, and another eleven had signs of middle ear inflammation on CT/MRI. Thus, a total of 66 patients (31%) were considered to have an otogenic meningitis. The number of otogenic meningitis cases in each age group are shown in Table 2. The proportion of otogenic meningitis cases was 25% (18/72) and 34% (48/142) in the pre- and post-PCV periods, respectively (*p* = 0.19), so there was no evidence of a change in the proportion of otogenic meningitis after the introduction of PCV. Patients with otogenic meningitis had higher odds of having a high fever on admission to hospital (OR 3.8; 95% CI 1.4–10.8; *p* = 0.01), but apart from that, there was no evidence of a different clinical presentation compared to those with meningitis of other origin. There was no association between death and otogenic meningitis. The responsible pathogen could be identified in 57 of the otogenic meningitis cases (86%). The most commonly identified pathogen in otogenic meningitis was *S. pneumoniae* (*n* = 51), followed by *S. pyogenes* (*n* = 3) and *H. influenzae* (*n* = 3). Interestingly, *N. meningitidis* was found in the cerebrospinal fluid in two cases of otogenic meningitis, both times together with *S. pneumoniae*. Patients with pneumococcal meningitis had four times the odds of their meningitis being otogenic (OR 4.1; 95% CI 2.1–8.0; *p* < 0.001), whereas patients with meningococcal meningitis were very unlikely to have a concurrent AOM (OR 0.17; 95% CI 0.04–0.74; *p* = 0.02). Patients with *S. pneumoniae* also had greater odds of having a high fever (OR 2.6; 1.2–5.9; *p* = 0.02). Those with *S. pyogenes* as the causative pathogen were few, and though the odds for them having a concurrent AOM was higher, this could easily have occurred by chance (OR 3.5; 95% CI 0.56–21.3; *p* = 0.18). We have reported elsewhere that otogenic meningitis was associated with an increased risk of hearing loss [6].

**Fig. 2:**
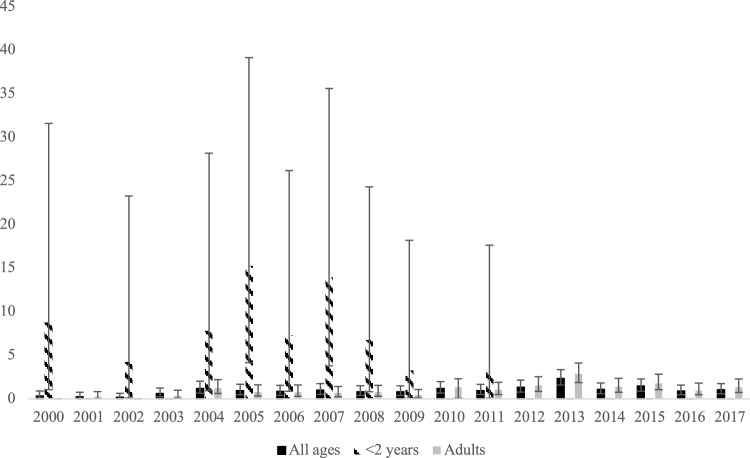
Yearly incidence of bacterial meningitis/100.000 inhabitants showing all ages, children < 2 years and adults (> 20 years) with 95% confidence intervals

The number of meningitis cases per year varied between four and 30; the overall average incidence varying between 0.17/100.000 and 2.35/100.000 inhabitants (Fig. 2). In the pre-vaccination period, children under the age of two accounted for 23% of the total number of meningitis cases, compared to just 1% in the post-vaccination period (Table 3). As shown in Table 4, one and three children in the two lowest age groups, respectively, had pneumococcal meningitis after the introduction of PCV. The one child in the lowest age group, had a non-vaccine serotype infection (serotype 33). As for the three cases in the age group 2–11 years, at least two children had not been vaccinated, as they were too old to be eligible for the vaccination programme. Vaccination status for the third child was not possible to conclude from the medical files, and the serotype was unknown.

**Table 3:** Proportion of meningitis cases per age group before and after the introduction of PCV; N (% of cases during the respective period)

Proportion of cases (all cause meningitis) per age group pre- and post-PCV		Age group					
		< 2	2–11	12–19	20–64	≥ 66	Total
*N* (%)	Pre-PCV*	17 (23.3)	4 (5.5)	8 (11.1)	26 (36.1)	17 (23.6)	73 (100)
	Post-PCV*	2 (1.4)	5 (3.5)	10 (7.0)	66 (46.5)	59 (41.6)	143 (100)

*Pre-PCV = 2000–2008; Post-PCV = 2009–2018

**Table 4:** Association between age and pneumococcal infection in the pre- and post-PCV periods. (Univariable logistic regression) N (% of cases in the respective age group)

*S. pneumoniae*		Age group					
		< 2	2–11	12–19	20–64	≥ 66	Total
Pre-PCV*	*N* (%)	15 (88%)	1 (25%)	0 (0%)	15 (58%)	8 (47%)	39 (53%)
	OR (95% CI)	Ref.	0.04 (0.003–0.66	–	0.18 (0.03–0.96)	0.12 (0.02–0.69)	
	p	–	0.02	–	0.045	0.017	
Post-PCV*	N (%)	1 (50%)	3 (60%)	2 (20%)	38 (57%)	35 (59%)	79 (55%)
	OR (95% CI)	Ref.	1.5 (0.06–40.6)	0.25 (0.01–6.0)	1.3 (0.08–21.8)	1.4 (0.09–24.5)	
	p	–	0.8	0.4	0.85	0.79	

*Pre-PCV = 2000–2008; Post-PCV = 2009–2018

On the other hand, all-cause meningitis cases increased in the adult population. In adults (the two upper age groups), the average incidence in the pre-vaccination period was 0.53/100.000 compared to 1.47/100.000 in the post-vaccination period, resulting in a net increase in the number of meningitis cases (Table 3, Fig. 2). The increased number of adult meningitis cases seemed not to be caused by one specific pathogen, as the proportions of positive cultures for each specific pathogen were similar before and after the introduction of PCV (data not shown), but rather by a general increase in the number of cases.

Administering antibiotics to patients as quickly as possible was often prioritised to the performance of a lumbar puncture, meaning that the responsible pathogen could only be identified in 177 of the patients (82%), however, in all paediatric cases. The type of pathogen varied between age groups (Table 2). By far the most common pathogen was *S. pneumoniae*, occurring in more than 50% of the cases (Tables 1 and 2). In the pre-vaccination period, there was strong evidence that *S. pneumoniae* was more common in the youngest age group (Table 4). There was also very strong evidence that *N. meningitidis* was more common among teenagers (Table 5).

**Table 5:** Association between age and meningococcal disease (Univariable logistic regression)

		Age group				
		< 2	2–11	12–19	20–65	> 66
N. meningitidis	OR (95% CI)	Ref.	4.25 (0.57–31.9)	14.6 (2.6–82.7)	0.8 (0.16–4.1)	0.11 (0.01–1.32)
	*p*	–	0.16	0.002	0.8	0.08

## Discussion

In this retrospective study on bacterial meningitis, performed during 18 years in Sweden’s third most populous region, 31% of all meningitis cases were of otogenic origin. Not many studies have addressed this question. A Portuguese regional retrospective study [7] on patients of all ages (= 201) with bacterial meningitis found a concurrent AOM in only 7% of the cases, whereas a national 2-year cohort of Danish patients with pneumococcal meningitis (*n* = 187) found that 30% had AOM [8]. A local study from the UK [9] retrospectively reviewed 87 adult cases with bacterial meningitis and found that 15% of the patients had AOM, which is in stark contrast to a local Israeli study (*n* = 45), where 58% of adult meningitis patients had AOM [10]. In several of these studies, it is not clear how many of the patients had undergone neither otoscopy nor CT, meaning there was no way of having any indication of whether there was a concurrent AOM. This, in turn, could mean that the proportion of otogenic meningitis was underestimated. The Danish and the English study were performed before the introduction of PCV, and the Israeli study before and after. In Portugal, PCV was readily available during the time of the study, but it was not included in the national vaccination programme. There was thus no clear association between PCV and the proportion of otogenic meningitis. According to data from the ECDC, IPD incidence in general vary between countries, with Portugal—with only a very small proportion of otogenic meningitis—showing the lowest incidences and the North European countries the highest, the incidences in e.g. Denmark and Sweden being very similar [11]. The only prospective study addressing the question was performed in Angolan children [12], who all children had their ears examined. These authors found that 62 of 512 children (12%) had concurrent AOM.

The Swedish national quality register for bacterial meningitis reports that about a third of meningitis cases have an infectious focus in the ears. However, just as in the studies referred to above, it is not clear from the register to what extent patients have had an ear examination [13]. The results in this study, where 99% of the patients had undergone at least one of otoscopy or imaging, agree well with the all-age Danish study and the paediatric Spanish study. Clinical experience as well as data from the present study indicate that, despite national guidelines stating that an ear examination should always be performed in meningitis patients, this is often not the case. In our study, only 116 of the 216 patients (54%) had any record of otoscopy or otomicroscopy. In the rest of the cases, our conclusion about otogenic origin was based on the findings on CT/MRI. In this way, we tried to estimate the proportion of meningitis cases originating from an AOM. However, the other side of the coin, i.e., how large a proportion of AOM cases develop meningitis is not possible to estimate from our data.

Animal research has shown that the development of hearing loss in pneumococcal meningitis is correlated to bacterial load in the middle ear [14]. This indicates that draining of a middle ear infection ought to be important in order to decrease the risk of hearing loss. This would, in turn, emphasise the importance of ear examination.

Not surprisingly, patients with otogenic meningitis were more likely to have a pneumococcal infection; however, there was no evidence of the proportion of otogenic meningitis declining after PCV, perhaps since the study was not powered to prove any such difference.

The overall mortality rate in this study was 12%, and the mortality rate for pneumococcal meningitis was 7%, which is somewhere in between mortality rates for pneumococcal meningitis reported from Germany (6%) [15], and England and Wales (18%) [16].

During the 18-year study period, meningitis incidence varied greatly from year to year, ranging from 0.18 to 2.35 cases/100.000 inhabitants. These incidences correspond well to previously reported figures from high-income countries [1].

In this study, bacterial meningitis almost disappeared among the youngest children after the introduction of PCV. Previous studies from various countries have also shown a sharp decrease in vaccine-type pneumococcal meningitis in children, particularly in those < 2 years of age, however, many have also noted a simultaneous increase in non-vaccine type meningitis, resulting in either a net increase or a non-significant decrease in net meningitis cases [17–22]. Two review articles from 2020 and 2021 have concluded that the promising decline in pneumococcal meningitis seen after the introduction of PCV was temporary, and that non-vaccine serotypes are a major challenge in the future [23, 24]. This study contains data until 2017, i.e. nine years after vaccine introduction in Sweden, but saw no tendency of an increased incidence in children, however, the findings from other countries highlight the importance of a continued surveillance.

The possible herd effect by PCV on pneumococcal meningitis in adults has been less well studied, but some authors have reported a decrease in incidence also among certain adult age groups [17, 18, 25]. Others, however, have reported a net increase in pneumococcal meningitis due infection with non-vaccine serotypes [26]. Though the number of cases in this study varied substantially from one year to another, the average incidence post-PCV was almost three times as high as pre-PCV, making a true increase highly likely. As serotyping was missing from the medical files in most cases in our study, we can draw no conclusions about serotype replacement. The fact that only two cases were noticed post-PCV in children < 2 years (in 2009 and 2011) is surprising if non-vaccine types had started to circulate, however, new, less invasive non-vaccine types might have been circulating. However, the fact that uncomplicated AOM continued to decrease in Sweden at least until 2014 [27] indicate that replacement with pathogenic serotypes had not yet occurred. A French study investigating the incidence of pneumococcal meningitis between 2001 and 2017, i.e. virtually the same period, found a decrease in children and elderly alike until 2015, after which incidences in both age groups rebounded in parallel due to serotype replacement [28].

Like previous studies [1, 24], the present confirmed *S. pneumoniae* followed by *N. meningitidis* as the major pathogens in bacterial meningitis. The same picture is seen in the Swedish quality register for bacterial meningitis [13]. During the pre-vaccination period, *S. pneumoniae* was most common among the very youngest, and in the post-vaccination period among adults. *N. meningitidis* was most prevalent among teenagers. This age distribution is well known [13, 29]. Conjugate meningococcal vaccines, which have proved to be very efficient in preventing meningococcal meningitis, have not been introduced in the Swedish vaccination programme.

Vaccination against *H. influenzae* type B was introduced in the Swedish vaccination programme in 1993. This pathogen only accounted for a few meningitis cases in this study; all but one in non-vaccinated adults.

In conclusion, this 18-year cohort from an entire Swedish county showed a dramatic decline in childhood meningitis after the introduction of PCV, but also signs of an increased incidence in adults during the same period. The fact that a third of the meningitis cases were of otogenic origin, but only half of the patients had their ears examined, emphasises the need for better compliance with national guidelines, which state that otoscopy should always be performed in patients with meningitis. It is important to continue to monitor what happens to meningitis incidences, and to closely monitor serotype shifts.

## Data Availability

The data that support the findings of this study are available from the corresponding author, [MGS], upon reasonable request.
